# Nutritional Deficiencies in Morbid Obese Patients Before and After Laparoscopic Sleeve Gastrectomy

**DOI:** 10.5152/tjg.2022.21935

**Published:** 2022-10-01

**Authors:** Ozan Şen, Dilara Çetin, Göknel Dumanlı, Ahmet Gökhan Türkçapar

**Affiliations:** 1 Türkçapar Bariatrics, Obesity Center, İstanbul, Turkey; 2 Nişantaşı University Faculty of Medicine, İstanbul, Turkey

**Keywords:** Bariatric surgery, obesity, sleeve gastrectomy, vitamin deficiency

## Abstract

**Background:**

This study aims to assess the prevalence of preoperative and postoperative nutritional deficiencies and associated factors in patients who are eligible for laparoscopic sleeve gastrectomy.

**Methods:**

Patients who underwent primary laparoscopic sleeve gastrectomy between December 2018 and April 2020 were included in the study. All patients were screened by detailed laboratory tests pre- and post-laparoscopic sleeve gastrectomy 6th and 12th months. Patients’ data, which were recorded prospectively, were analyzed retrospectively.

**Results:**

A total of 228 patients were included in the study. The mean age was 39 ± 11.5 (60% female), and the mean body mass index was 41.2 ± 6.3 kg/m^2^. In the preoperative period, anemia was detected in 20 female patients (9%), low ferritin levels were detected in 25%, B12 and folic acid deficiencies were detected in 2.6% and 12.3%, respectively, and vitamin D deficiency was detected in 76% of the patients. During the postoperative follow-up, 77% of the patients received multivitamin supplements regularly. Mean body mass index regressed to 27.1 ± 4.2 kg/m^2^ in the first year. Incidence of anemia was found at 4.8%, low ferritin levels were 14%, folate deficiency was 5.3%, B12 deficiency was 5.3%, and vitamin D deficiency was 25% in the 12th month. Vitamin A, zinc, biotin, and thiamine deficiencies were 8.8%, 6.6%, 11%, and 2.2% in the 12th month, respectively.

**Conclusion:**

In the preoperative period, we detected significant deficiencies in some vitamins. The incidence of de novo vitamin ­deficiency during post-laparoscopic sleeve gastrectomy follow-up was low. Regular multivitamin–multimineral use may have an effect on this.

**Main Points**

- Nutritional deficiencies are seen more frequently in people suffering from obesity.
- There is an ongoing debate in the literature on nutritional deficiencies after laparoscopic sleeve gastrectomy (LSG).
- In our study, we detected significant deficiencies in some vitamins before and after LSG.

## Introduction

Bariatric surgery is the most effective method in combating obesity.^1,2^ Bariatric surgical procedures are distinguished as malabsorptive and restrictive methods or combination of them. Among restrictive methods, laparoscopic sleeve gastrectomy (LSG) has become the most preferred method on a global scale.^3^ Nutritional deficiencies are seen more frequently in people suffering from obesity.^4,5^ Pre-existing vitamin deficiencies may worsen, and new vitamin deficiencies may develop in some patients following bariatric surgery.^6,7^

While some studies indicate that post-LSG vitamin deficiency is not common,^8^ other studies report significant post-LSG nutritional deficiencies.^9,10^ This study aims to show pre- and postoperative nutritional deficiencies and associated factors in patients who are eligible for LSG.

## Materials and Methods

This study was approved by the Institutional Ethics Committee (ATADEK-2020/26). All patients were informed about the study in detail, and written consents were obtained. A total of 250 patients who underwent primary LSG between December 2018 and April 2020 and completed 1 year of follow-up were included in the study. Our preoperative workup protocol and surgical technique have been previously described.^11^ All the patients were screened by detailed laboratory tests with respect to metabolic parameters and vitamin deficiencies. Surgery was not postponed in patients in whom vitamin deficiency or mild anemia was detected; however, the required treatment targeting the relevant vitamin was initiated immediately. World Health Organization (WHO) guidelines have been followed for the definition of anemia. Patients with hemoglobin (Hb) below 10 mg/dL were excluded from this study. All LSG operations were done by the same team and the same technique.^11^

### Postoperative Medications and Follow- Up

Postoperatively, all patients were prescribed enoxaparin sodium (clexane, 40 or 60 mg/day, Sanofi-Aventis, Turkey) to prevent venous thrombosis for a duration of 1 week, Proton Pump Inhibitor (PPI) (Nexium, 40 mg/day, Astra Zeneca, Turkey) for a duration of 6 months, and multivitamin supplement (Barifit tablet/day) for a duration of 1 year. The composition of the multivitamin (mv)-multimineral (mm) supplements is in accordance with the American Society for Metabolic and bariatric Surgery guidelines and has been previously shown^11,12^ (Table 1). Also, all patients were prescribed ursodeoxycholic acid (Ursactive, Pharmactive, Turkey) 500 mg/day for a duration of at least 6 months to reduce the risk of gallstone formation due to postoperative weight loss.^13^ In addition, patients with severe vitamin D deficiency were treated with 50 000 IU per week oral vitamin D for 1 month (Devit-3 drop form, Deva, Turkey), then 50 000 IU monthly thereafter. Vitamin D supplementation was recommended to all patients following their surgery. We do not recommend extra B12 vitamin routinely except for the amount that is in the Barifit multivitamin (350 µg B12) after LSG.

Patients were re-evaluated at post-LSG at 6th^h^ and 12th months in terms of weight loss outcomes and laboratory tests. In the preoperative period, all patients were monitored for Hb, iron, ferritin, B12, folic acid, and vitamin D levels. In the postoperative period, vitamin A, thiamine, biotin, and zinc levels were also monitored. All tests were run in the same laboratory. In all patients, identical reference values were used to monitor vitamin levels, and values below these reference values were defined as vitamin deficiency (see Table 3). Patients’ data, which were recorded prospectively, were analyzed retrospectively. Patients’ weight loss outcomes, mv-mm supplement usage, and the prevalence of vitamin deficiency were evaluated in the preoperative and post-LSG follow-up period.

### Statistical Analysis

Statistical analysis was performed using Statistical Package for the Social Sciences (version 21, IBM Corp.; Armonk, NY, USA). Standard deviation and mean values were used for the variables with normal distribution, and median values were used for the variables that were not normally distributed. Chi-square or Fisher’s exact tests were used for categorical variables. *P* < .05 were considered statistically significant.

## Results

A total of 228 patients who underwent primary LSG and completed 1-year follow-up were included in the study; 22 patients were excluded from the study due to a lack of blood tests during the postoperative follow-up period. The mean age of the patients was 39 ± 11.5 (60% females) and the mean body mass index (BMI) was 41.2 ± 6.3 kg/m^2^. At baseline, 78%, 11%, 27%, 49%, and 25% of the patients had insulin resistance, type-2 diabetes mellitus, hypertension, hyperlipidemia, and sleep apnea, respectively. Anemia was detected in 20 patients (9%) in the preoperative period. All of them were females and ferritin levels were low in 95% of them. In the preoperative period, low iron (<60 µg/dL) and ferritin (<30 ng/mL) levels were detected in 25% of the patients. B12 and folic acid deficiencies were detected in 2.6% and 12.3% of the patients, respectively. Significant vitamin D deficiency (<30 ng/mL) was detected in 76% of the patients. It was observed that vitamin D deficiency in the high BMI group (BMI > 40 kg/m^2^, 85% ) was a little bit higher than the other group (BMI < 40 kg/m^2^, 78%) (*P* = .04).

During the post-LSG period, mean BMI regressed to 29.5 ± 4.7 kg/m^2^ in the sixth month and 27.1 ± 4.2 kg/m^2^ in the first year. The percentage of mean excess weight loss was found to be 79% ± 21% and 94% ± 24% in the sixth month and first year, respectively. Preoperative demographics and postoperative weight loss outcomes are shown in Table 2. Two patients had postoperative bleeding. One of them was treated conservatively. The other patient was re-operated. She had a diffuse intra-abdominal hematoma. No active bleeding was detected. These 2 patients were excluded from the study due to anemia caused by postoperative bleeding after surgery. No other complications were observed.

**Figure 1.**
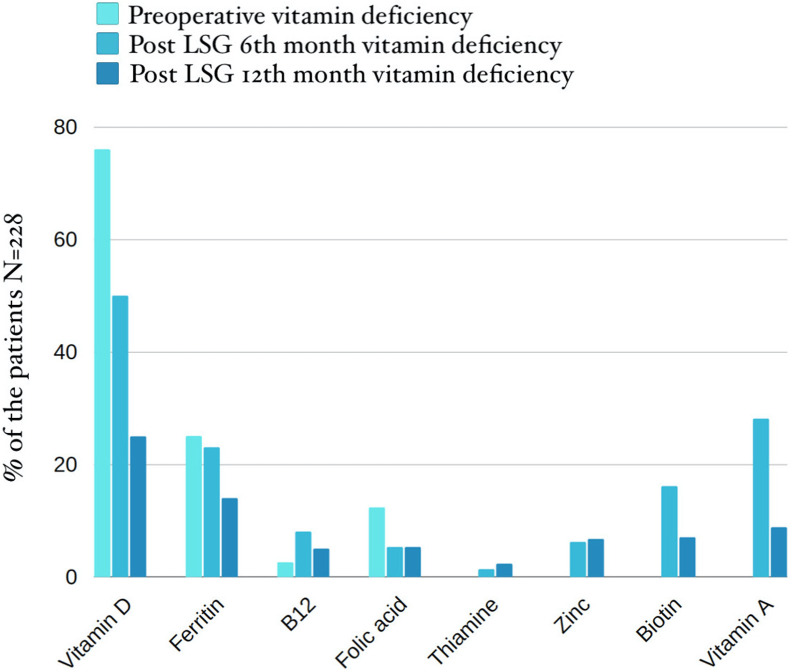
Pre- and post-LSG vitamin deficiencies. LSG, laparoscopic sleeve gastrectomy.

Laboratory tests were repeated at the 6th and 12th months post-LSG. The incidence of postoperative anemia was 6% and 4.8% in the 6th and 12th months, respectively. All of these patients were females and 60% of them had anemia in the preoperative period as well. Low ferritin levels were detected in 23% and 14% of the patients in the 6th and 12th months, respectively. It was observed that approximately 75% of these patients had low ferritin levels in the preoperative period as well. The incidence of folate deficiency in different patient groups was 5.3% and 5.3% in the 6th and 12th months, respectively, and B12 deficiency was 8% and 5% in the 6th and 12th months, respectively. Additionally, the incidence of vitamin D deficiency was 50% and 25% in the 6th and 12th months, respectively. Other vitamin deficiencies showed the following rates for vitamin A, zinc, biotin, and thiamine deficiency, respectively: 28%, 6.1%, 16%, and 1.3% in the 6th month and 8.8%, 6.6%, 7%, and 2.2% in the 12th month. Vitamin deficiencies, which were observed in the preoperative and post-LSG follow-up period, are summarized in Figure 1 and Table 3. During the post-LSG 1-year follow-up, it was observed that 175 patients (77%) received mv-mm supplements regularly and 23% of the patients told they did not receive mv-mm supplements regularly.

## Discussion

Vitamin deficiency is common in people suffering from obesity.^5,14,15^ Eating high-calorie and high-fat diets that restrict some nutrients may be one of the reasons. One of the most significant outcomes of micronutrient deficiency is iron deficiency characterized by low Hb levels. The most significant marker of iron deficiency is the serum ferritin level. Serum iron level alone is not a sufficient indicator for iron deficiency. In our study, iron deficiency anemia was detected in 9% (n = 20) of the patients in the preoperative period. Low ferritin and iron levels were also detected in 25% of the patients irrespective of Hb levels. Oral iron supplements with a prophylactic dosage (1 tablet/day) were initiated in the early period in patients with iron deficiency.

During the post-LSG follow-up period, the incidence of anemia was observed at 6% and 4.8% in the 6th and 12th months, respectively. In the sixth month post-LSG, 23% of the patients still had low ferritin levels. It was observed that 60% of these patients had ferritin deficiency in the preoperative period as well. Despite oral replacement therapy, ferritin levels did not increase in the majority of these patients during the follow-up period. Decreased stomach acidity in the post-LSG period along with the PPI treatment for 6 months may have caused a bioavailability problem with oral iron preparations.^16,17^ This was considered as a possible reason for unsuccessful oral iron treatment, which was initiated in the early period, in majority of the patients. Therefore, we have now decided to limit the use of PPI as much as possible after LSG (2 months). Additionally, our primary preference now is intravenous replacement therapy in patients with iron deficiency during the pre-LSG period. In the literature, the rate reported for iron deficiency is 17%-44% in the post-LSG period.^15,18^ In our study, the rate of de novo low ferritin incidence at the end of the first year was 4.8%, which is lower than that reported in the literature. One of the reasons for this may be the use of regular mv-mm supplements by majority of the patients after LSG.

Other common vitamin deficiencies in individuals with obesity are B12 and folic acid. While B12 is an important vitamin in terms of DNA synthesis and neurological functions, folic acid is also important for DNA synthesis and repair, as well as for red blood cell formation. The absorption of vitamin B12 requires intrinsic factor secreted from parietal cells in the stomach. In the literature, the rates reported for B12 and folic acid deficiencies are 2%-18% and up to 50%, respectively.^15^ In our study, the incidence of B12 and folic acid deficiencies were 2.6% and 12.3%, respectively, in the preoperative period. Additionally, at the end of the first follow-up, rates for de novo B12 and folic acid deficiencies were 3.5% and 3%, respectively. In the literature, the rates reported for post-LSG B12 and folic acid deficiencies are 4%-20% and up to 65%, respectively.^15^ In our study, this ratio was also lower than that reported in the literature.

The most frequent vitamin deficiency in obese population is vitamin D. Vitamin D is important as it is involved in various mechanisms such as intestinal calcium absorption, immune system, wound healing, and cell differentiation. Numerous studies have shown that vitamin D deficiency may be seen at rates up to 90% in people suffering from obesity and also in the post-bariatric follow-up period.^15,19,20^ In our study, vitamin D deficiency was detected in 76% of the patients in the preoperative period. This rate decreased to 50% in the 6th month and 25% in the 12th month. Since patients were encouraged to receive regular vitamin D during the follow-up period, the rate of new deficiencies was very low (Figure 1 and Table 3). Another reason for this may be due to weight loss after the surgery.

Chronic thiamine deficiency in the post-bariatric surgery period may cause severe neurological deficits.^21^ In our study, thiamine deficiency was detected in 1% (n = 3) of the patients in the 6th month and in 2% (n = 5) of the patients in the 12th month during the post-LSG follow-up period. None of these patients were symptomatic. Supportive treatment was started in these patients in the early period. Post-laparoscopic sleeve gastrectomy thiamine deficiency may develop in those who are undernourished or due to excessive consumption of alcohol. In a previous study, we have also shown that post-LSG alcohol consumption increased significantly in some patients.^22^

In our study, vitamin A deficiency was present in 28% of the patients at the 6th-month post-LSG. With oral vitamin A replacement therapy, this rate decreased to 8.8% in the 12th month. Vitamin A deficiency may be seen at a rate of 14% in those suffering from obesity and at rates up to 70% after surgery such as gastric bypass and duodenal switch.^14^ One study has demonstrated the rate of post-LSG vitamin A deficiency as 55%.^23^ Since we did not look at preoperative vitamin A levels, we do not know if they are a pre-existing or newly occurring deficiency.

Among other significant vitamin deficiencies in the post-bariatric surgery period are biotin and zinc deficiencies. In our study, zinc and biotin deficiency rates were 6.6% and 7%, respectively, in the 12th month. These 2 vitamins are important for healthy skin and hair. In particular, post-LSG hair loss is associated with a lack of either of these vitamins. In one of our studies, we have shown a rate of 70% for post-LSG hair loss and that the use of biotin is not significantly effective on hair loss.^11^

Our study has some limitations. First of all, this is a single-center, retrospective study. Moreover, our sample size is small and includes only 1-year results for follow-up. We did not examine thiamine, zinc, biotin, and vitamin A levels in the preoperative period. Therefore, we were unable to assess if deficiencies associated with these vitamins, which we detected during the follow-up period, were already present in the baseline or developed later on. We questioned our patients only in terms of whether they used mv-mm supplement regularly during the 1-year follow-up. We did not examine the types of diets followed by our patients in detail. In our study, the incidence rate for de novo vitamin deficiency during the post-LSG follow-up period was lower than that reported in the literature. We believe that regular mv-mm supplement use by patients had an effect on this; however, weight loss or the type of diet followed by patients may also have had an impact. It is difficult to make a definite assertion on this. Further larger-scale studies are needed on this subject.

## Conclusion

In our study, we analyzed patient data in terms of pre- and post-LSG vitamin deficiency. In the preoperative period, we detected significant deficiencies in some vitamins, particularly in vitamin D. We demonstrated that vitamin deficiency detected in the preoperative period did not improve easily during the follow-up period despite regular use of mv-mm supplement and specific oral replacement therapy. Factors that decrease post-LSG oral bioavailability may have had an impact on this. Therefore, prioritizing other replacement methods over oral replacement therapy may be a more adequate approach for patients with post-LSG vitamin deficiency.

## Figures and Tables

**Table 1.** Composition of the Multivitamin Supplement

	Dose	Percent Recommended Daily Allowance
Vitamin A	1000 µg	125
Thiamine (vitamin B1)	1.5 mg	136.36
Riboflavin (vitamin B2)	1.7 mg	121.42
Niacin (vitamin B3)	30 mg	187.5
Pantotenic acid (vitamin B5)	10 mg	166.66
Vitamin B6 (pridoxine HCL)	4 mg	285.71
Folic acid	400 µg	200
Vitamin B12 (cobalamin)	350 µg	14 000
Vitamin C	60 mg	80
Vitamin D	25 µg	500
Vitamin E	15 mg	125
Biotin	600 µg	1.200
Iodine	150 µg	100
Iron	14 mg	100
Copper	1000 µg	100
Magnesium	60 mg	16
Zinc	15 mg	150
Chromium	120 µg	300
Molybdenum	200 µg	400
Folic acid	400 µg	200
Selenium	70 µg	127.27

HCL, hydrochloric acid.

**Table 3.** Preoperative and Postoperative Vitamin Deficiencies

n = 228	Anemia (Hb); <11.7 Female, <13.2 Male	Ferritin < 30 ng/mL	Iron < 60 µg/dL	Vitamin D < 30 ng/mL	B12 < 200 pg/mL	Folic acid < 3 ng/mL	Thiamine < 35 µg/dL	Zinc < 70 µg/dL	Biotin < 200 ng/L	Vitamin A < 300 µg/L
Preoperative assessment, n (%)	20 (9)	57 (25)	54 (24)	174 (76)	6 (2.6)	28 (12.3)	N/A	N/A	N/A	N/A
Post-LSG 6th month deficiency, n (%)	14 (6)	52 (23)	48 (21)	113 (50)	18 (8)	12 (5.3)	3 (1.3)	14 (6.1)	36 (16)	64 (28)
Sixth month De novo deficiency, n (%)	3 (1.3)	11 (4.8)	25 (11)	12 (5)	16 (7)	5 (2)	N/A	N/A	N/A	N/A
Post-LSG 12th-month deficiency, n (%)	11 (4.8)	32 (14)	15 (7)	58 (25)	11 (5)	12 (5.3)	5 (2.2)	15 (6.6)	16 (7)	20 (8.8)
12th month de novo deficiency n (%)	4 (1.7)	11 (4.8)	7 (3)	9 (4)	8 (3.5)	7 (3)	N/A	N/A	N/A	N/A

LSG, laparoscopic sleeve gastrectomy; N/A, not applicable; n, number.

**Table 2.** Patient Demographics and Weight Loss Outcomes

Men/women, n (%)	138/90
LSG (n)	228
Mean age (years)	39 ± 11.5
Baseline mean BMI (kg/m^2^)	41.2 ± 6.3
Baseline excess weight (kg)	44.7 ± 18.3
Sixth month mean BMI (kg/m^2^)	29.5 ± 4.7
Sixth month excess weight loss (%)	79 ± 21 (40-168)
First year mean BMI (kg/m^2^)	27.1 ± 4.2
First-year excess weight loss (%)	94 ± 24 (40-168)

LSG, laparoscopic sleeve gastrectomy; BMI, body mass index; n, number.
